# Supplementary material for: Solvent-Free Mechanochemical Synthesis of ZnO Nanoparticles by High-Energy Ball Milling of ε-Zn(OH)2 Crystals
Source: Nanomaterials (Basel). 2021 Jan 18;11(1):238. doi: 10.3390/nano11010238 (PMC7831064; doi:10.3390/nano11010238)
Supplement: Supplementary file 1 [file nanomaterials-11-00238-s001.pdf]

Supplementary Materials:

Solvent-Free Mechanochemical Synthesis of ZnO Nanoparticles by High-Energy Ball Milling of ε-$Zn(OH)_2$ Crystals

Gil Otis, Michal Ejgenberg and Yitzhak Mastai

Table of Contents

Description	Page no.
Characterization methods	**2**
**Figure S1** HR-SEM image of as synthesized ε-$Zn(OH)_2$ crystals	**2**
**Figure S2** XRD instrument peak width calibration	**3**
**Figure S3** WH-plots of milled powders	**3**
**Figure S4** ZnO polycrystalline aggregate size calculated using DLS	**4**
**Figure S5** Tauc plots for direct band gap calculation of milled NPs	**5**
**Figure S6** TEM images of 2 Cycles	**5**
**Figure S7** TEM images of 5 Cycles	**6**
**Figure S8** TEM images of 8 Cycles	**6**
**Figure S9** FTIR spectra of milled powders	**7**
**Figure S10** Beer lambert calculations for methyl orange (MO)	**7**

Characterization methods

High-resolution scanning electron microscopy (HR-SEM) images of the ε-$Zn(OH)_2$ crystals were taken using a field-emission, FEI, Helios 600 HR-SEM. Samples were sputtered with a 3 nm layer of iridium in order to reduce charging effects. Reflectance spectra of the powders was measured using a Cary 500 Scan UV-Vis spectrophotometer equipped with a diffused reflection UV (DRUV) solid sample holder. Dynamic light scattering (DLS) measurements were carried out using a Malvern Zetasizer 3000 H AS. All powder samples were suspended in double-distilled water (0.1 mg/mL), bath sonicated for 10 min, and then measured ten times. FTIR spectra were collected using a Thermo Scientific Nicolet iS10 FTIR spectrometer equipped with a Smart iTR attenuated total reflectance (ATR) sampler containing a single bounce diamond crystal. Data were collected and analyzed using OMNIC software. Spectra were collected in the 650–4000 cm^{-1} range at a spectral resolution of 4.

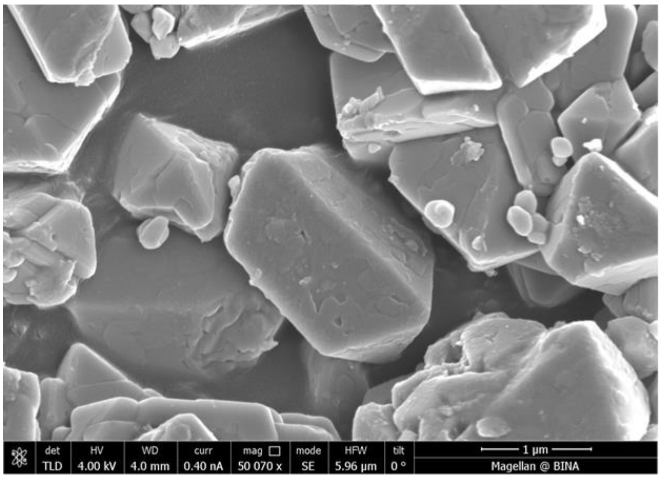

Figure S1: HR-SEM image of as-synthesized-$Zn(OH)_2$ crystals – average size of 2.4 ± 0.3 um.

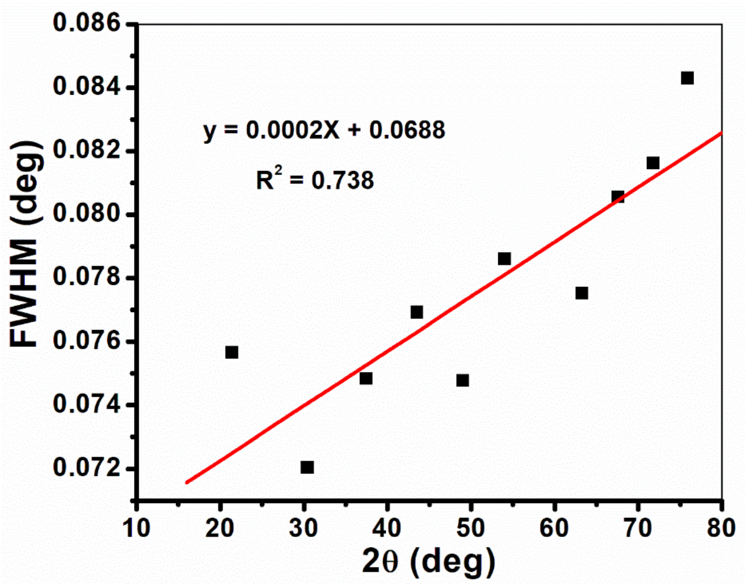

Figure S2: XRD instrument peak width calibration using LaB6 standard.

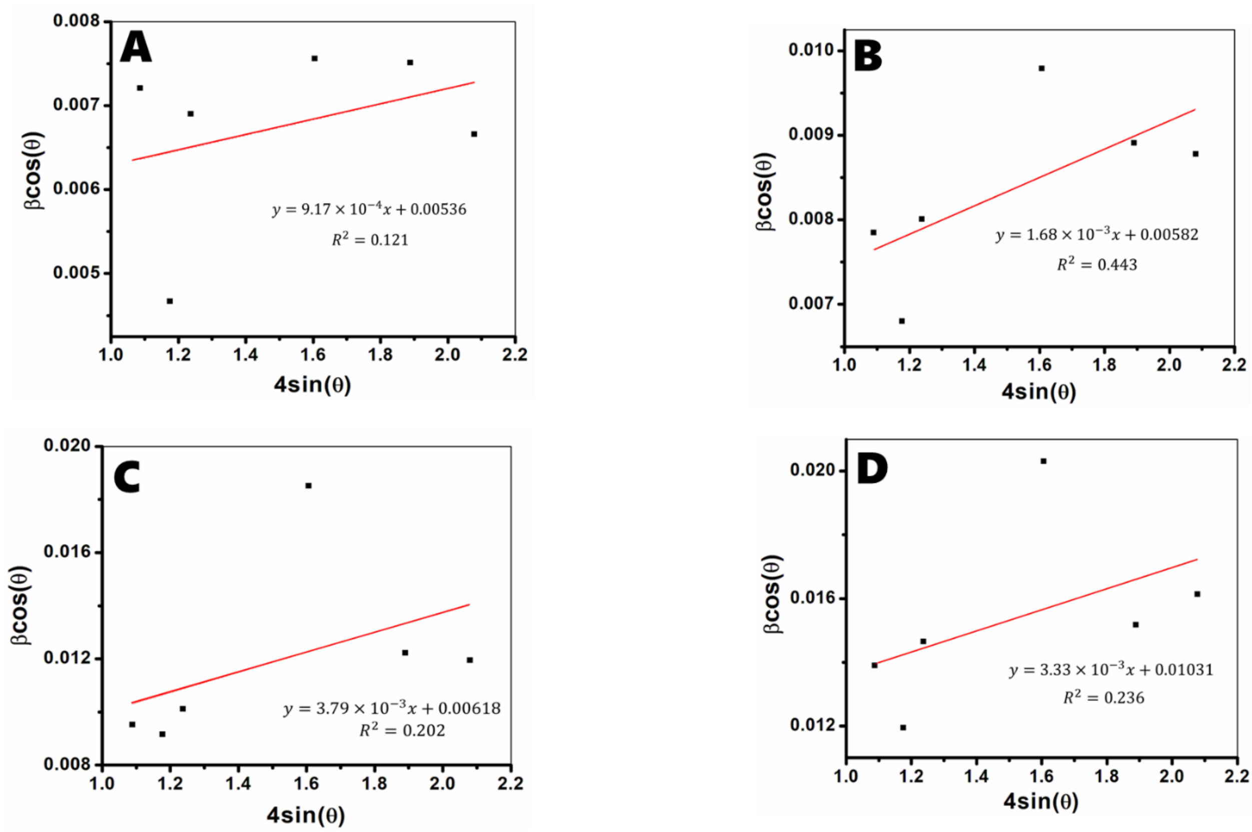

Figure S3: WH-plots of millied powders – 4sin(θ) versus cos(θ) for the six dominant diffraction ZnO peaks in 2 Cycles (**A**), 5 Cycles (**B**), 8 Cycles (**C**) and 10 Cycles (**D**).

Aggregate size was measured by DLS; powders were suspended in DDW and measured 10 times. The average particle size in solution (nm) and relative abundance of each size are presented in Figure S8. The DLS graph of 2 Cycles (red line) presents one large peak centered at around 1,000 nm and a much smaller peak centered around 250 nm; the large peak corresponds to residues of ε-$Zn(OH)_2$ in the sample, as confirmed by XRD and TEM, and the small peak, with a much lower abundance in the sample, fits a small ZnO nanocrystalline aggregate, which is smaller than all other products due to the smaller number of ZnO nanocrystals compared to the other samples.

5 Cycles also presents two peaks in the DLS graph, one centered at around 1,300 nm and the other centered around 490 nm; in this case, the large aggregates do not correspond to residues of ε-$Zn(OH)_2$, as no diffraction peaks that fit ε-$Zn(OH)_2$ were detected by XRD, however large quantities of a-$Zn(OH)_2$ were detected with TGA analysis, therefore we believe that the large peak has to do with the presence of large ZnO/a-$Zn(OH)_2$ aggregates that later break down with the milling process. As for the 8 and 10 Cycle samples, we only find one peak in DLS, meaning that all of the aggregates in the sample are of relatively the same size, however the 10 Cycles sample presents a much sharper peak in comparison to 8 Cycles, which means that it has a much narrower size distribution and a higher homogeneity. The 8 Cycles sample also contains a certain amount of a-$Zn(OH)_2$, so the presence of a small amount of ZnO/a-$Zn(OH)_2$ aggregates could explain the wider distribution of sizes. Altogether, from the DLS results we learn that throughout the transition from ε-$Zn(OH)_2$ to a-$Zn(OH)_2$, and finally to ZnO nanoparticles, the aggregate size decreases until it collapses to an average size of 500 ± 100 nm with a narrow size distribution.

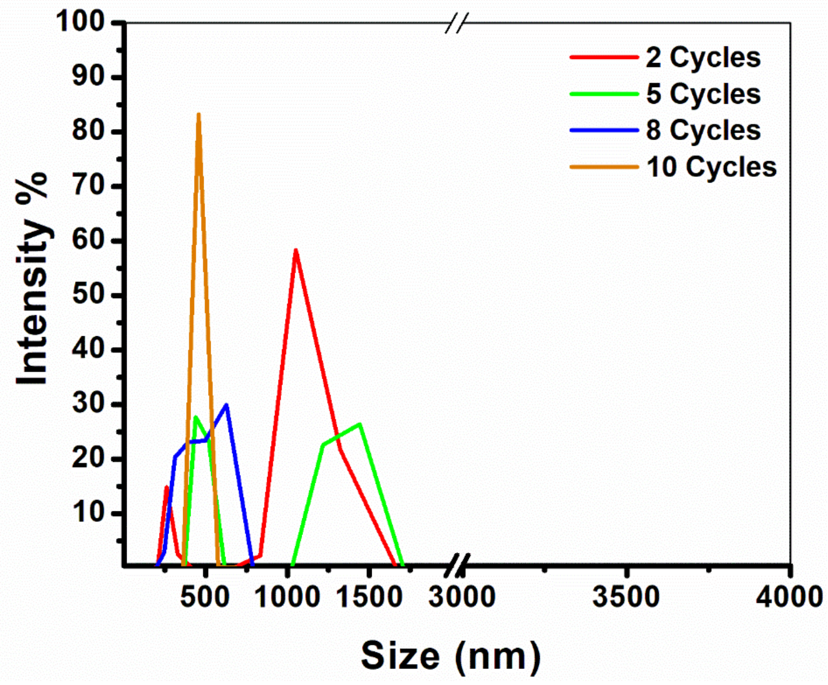

Figure S4: ZnO polycrystalline aggregate size calculated using DLS.

The band gap of the milled powders was examined by diffused reflection UV (DRUV) using the Kubelka–Munk (KM) method. The relationship between the scattering and adsorption of a direct band gap semiconductor is as follows: [1,2]

$$F(R_\infty) = \frac{K}{S} = \frac{(1 - R_\infty)^2}{2R_\infty}$$

Where $F(R_\infty)$ is the KM function assuming measured reflectance of an ideal infinitely thick specimen, K is the adsorption coefficient, and S is the scattering coefficient of the sample. The KM function could be used to calculate the band gap of the semiconductor by placing $F(R_\infty)$ instead of α in the Tauc equation (2) and plotting $(\alpha h\nu)^2$ versus energy

(eV). The region in the graph that shows a steep, linear increase of light absorption with increasing energy is characteristic of semiconductor materials, and the *x*-axis intersection point of the linear fit of the Tauc plot gives an estimate of the band gap energy.(2) For all powders, the band gap was found to be around 3.22 eV, regardless of the number of milling cycles, probably because the crystalline size in all of the samples is of the same order, therefore the minor changes in size did not have an effect on the electrical properties. The results are presented in Figure S5.

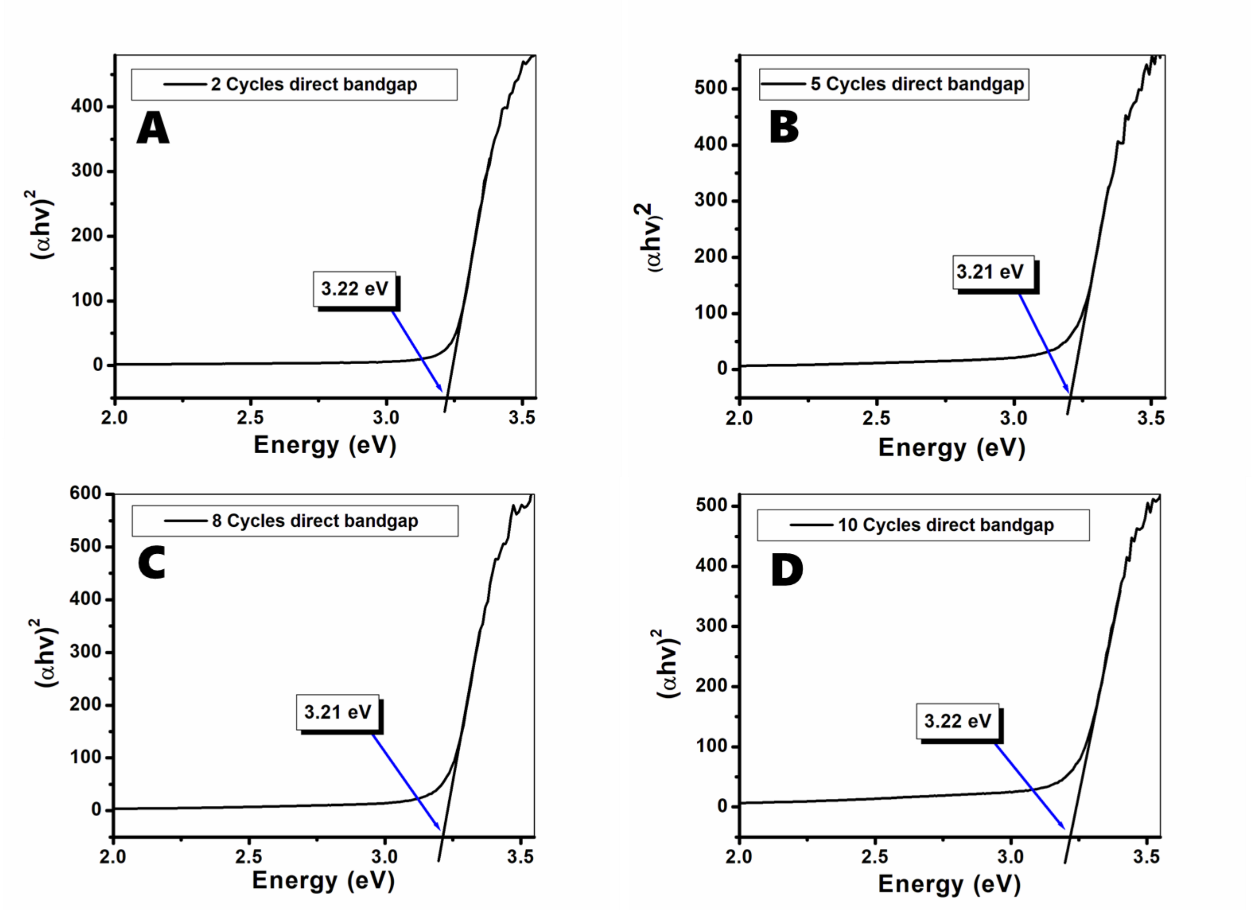

Figure S5: Tauc plots for calculation of direct band gaps of milled ZnO nanoparticles – $(hv)^2$ versus energy converted from reflection measurements of 2 (**A**), 5 (**B**), 8 (**C**) and 10 (**D**) cycles.

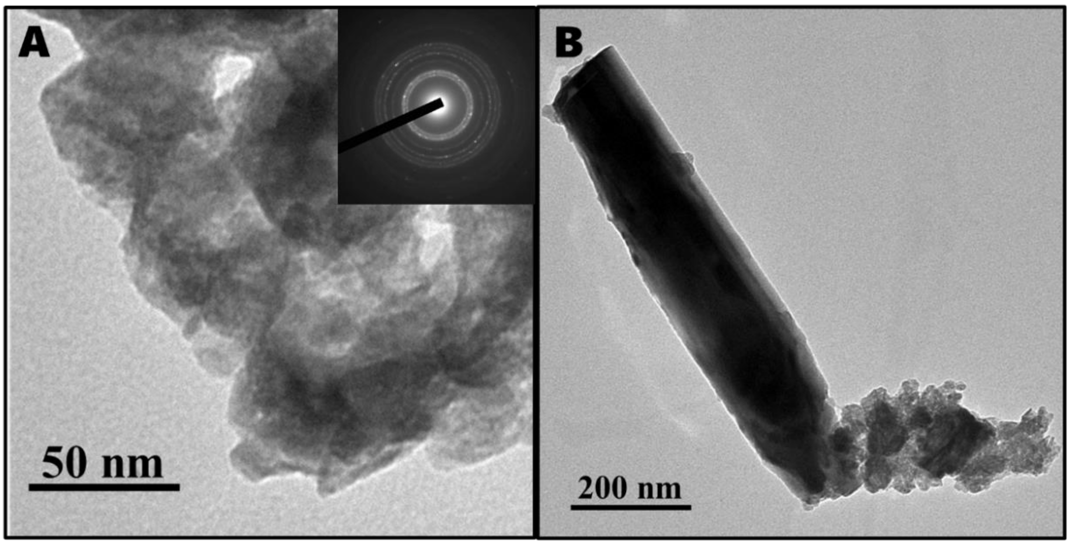

Figure S6: TEM images of 2 Cycles – **A.** Close-up of ZnO nanoparticle aggregate with diffraction pattern (inset). **B.** ZnO nanoparticles breaking from a large ε-$Zn(OH)_2$ crystal fragment.

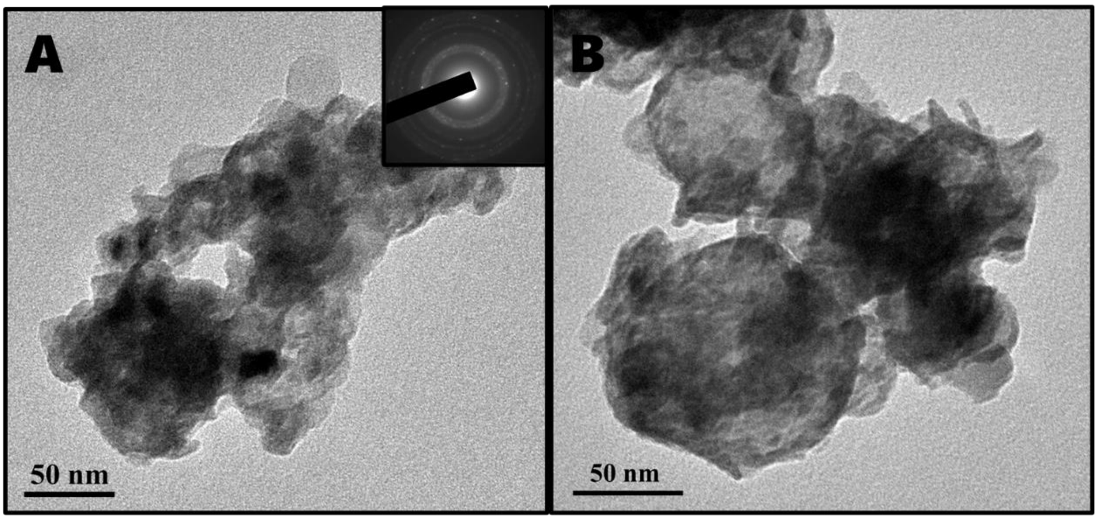

Figure S7: TEM images of 5 Cycles – **A.** Close-up of ZnO nanoparticle aggregate with diffraction pattern (inset). **B.** ZnO nanoparticles displaying larger aggregates (roughly 100 nm).

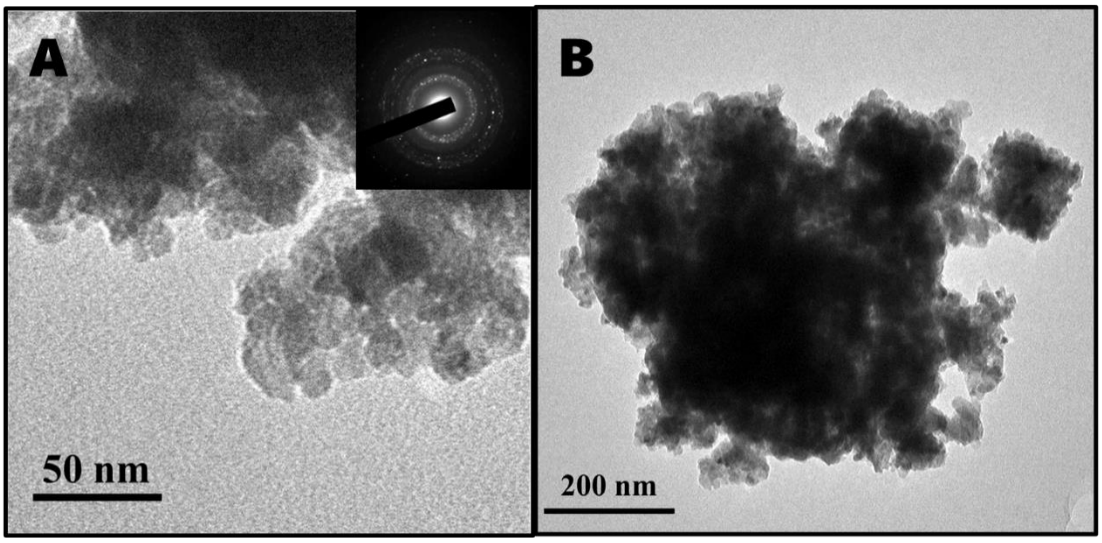

Figure S8: TEM images of 8 Cycles – **A.** Close-up of ZnO nanoparticle aggregate with diffraction pattern (inset). **B.** ZnO nanoparticles displaying larger aggregates (roughly 500 nm).

The structure of the milled powders was also investigated by Fourier transform infrared (FTIR) spectroscopy. All spectra, shown in Figure S9, show a wide peak at 3352 cm^{-1} in the characteristic area of OH stretching vibrations (3000–3500 cm^{-1}). We believe that this peak could be attributed to a-$Zn(OH)_2$, which is known to exist in all samples from the TGA analysis (Figure 4). In the 10 Cycles sample, the peak intensity decreased to a large extent, and the peak shifted to 3395 cm^{-1}, which is characteristic to water/ethanol molecules adsorbed onto ZnO [3]. In addition to the peaks

at the 3000–3500 cm⁻¹ region, a very small peak at 2975 cm⁻¹ corresponds to C–H stretching of an alkane, and two additional peaks at 1508 and 1382 cm⁻¹ correspond to C–O stretching and O–H bending [4,5]. This data is consistent with ethanol molecules that are adsorbed in the milled powders.

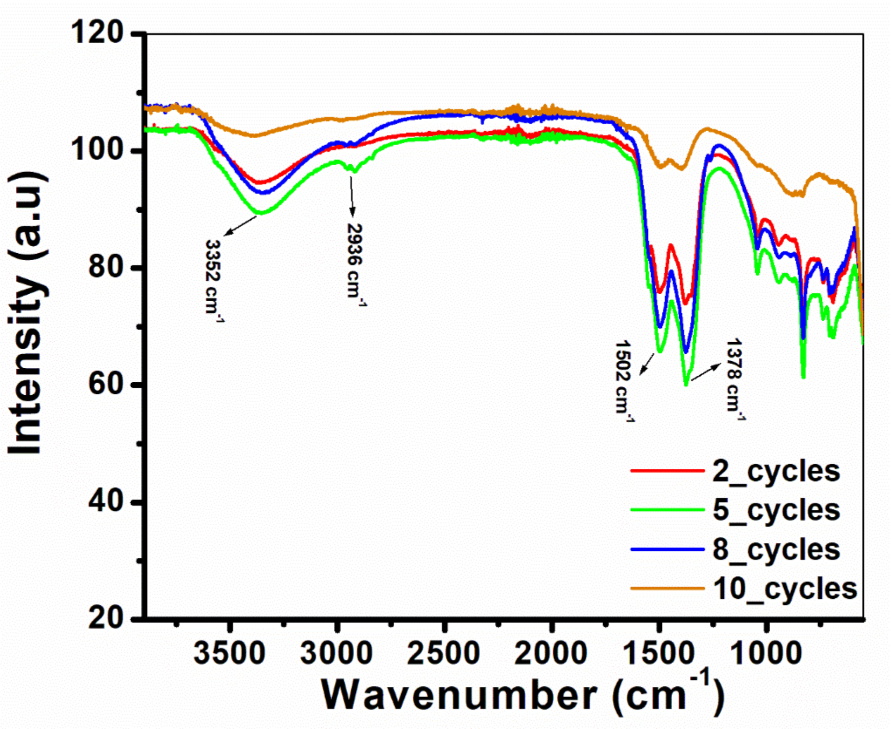

Figure S9: FTIR spectra of milled powders.

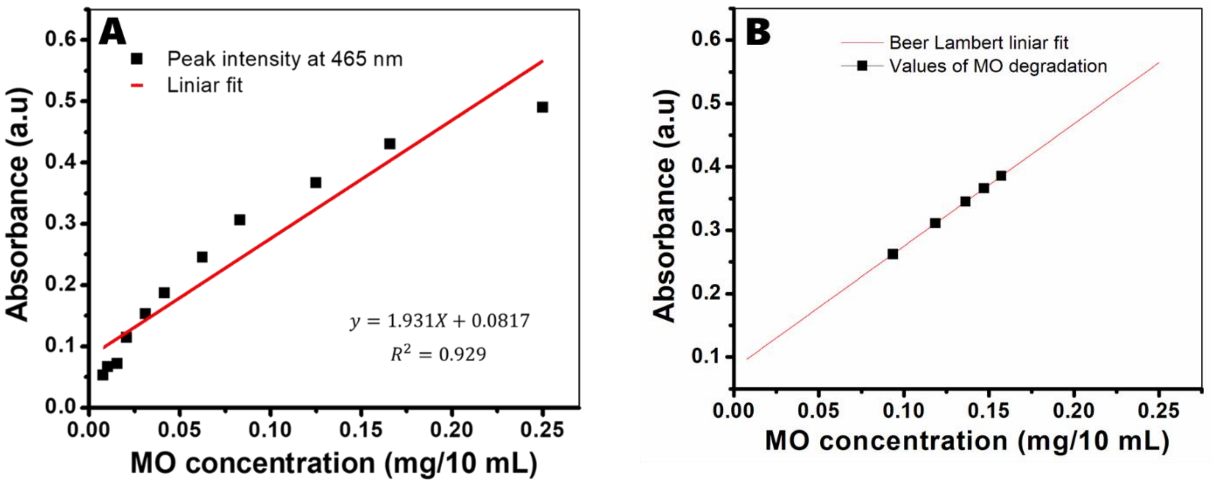

Figure S10: Beer Lambert calculations for methyl orange (MO). **A.** Calibration of MO absorbance at maximum intensity (at 465 nm). **B.** Calculation of dye concentration of the catalytically degraded MO using the absorbance at 465 nm.

References

1. Torrent J, Barrón V. Diffuse reflectance spectroscopy. Methods of Soil Analysis Part 5—Mineralogical Methods. 2008;5:367–85.

2. Makuła P, Pacia M, Macyk W. How to correctly determine the band gap energy of modified semiconductor photocatalysts based on UV–Vis spectra. ACS Publications; 2018.
3. Winiarski J, Tylus W, Winiarska K, Szczygieł I, Szczygieł B. XPS and FT-IR characterization of selected synthetic corrosion products of zinc expected in neutral environment containing chloride ions. Journal of Spectroscopy. 2018;2018.
4. Sood S, Kumar A, Sharma N. Photocatalytic and antibacterial activity studies of ZnO nanoparticles synthesized by thermal decomposition of mechanochemically processed oxalate precursor. ChemistrySelect. 2016;1(21):6925–32.
5. Balogun SW, James OO, Sanusi YK, Olayinka OH. Green synthesis and characterization of zinc oxide nanoparticles using bashful (Mimosa pudica), leaf extract: a precursor for organic electronics applications. SN Applied Sciences. 2020;2(3):1–8
